# Complement and *Chlamydia psittaci*: Early Complement-Dependent Events Are Important for DC Migration and Protection During Mouse Lung Infection

**DOI:** 10.3389/fimmu.2021.580594

**Published:** 2021-03-09

**Authors:** Martin Kohn, Christian Lanfermann, Robert Laudeley, Silke Glage, Claudia Rheinheimer, Andreas Klos

**Affiliations:** ^1^Medical School Hannover, Institute of Medical Microbiology and Hospital Epidemiology, Hannover, Germany; ^2^Medical School Hannover, Institute for Laboratory Animal Science, Hannover, Germany

**Keywords:** complement, *Chlamydia*, intracellular, adaptive immunity, C3a, dendritic cells, cobra venom factor

## Abstract

The zoonotic intracellular bacterium *Chlamydia psittaci* causes life-threatening pneumonia in humans. During mouse lung infection, complement factor C3 and the anaphylatoxin C3a augment protection against *C. psittaci* by a so far unknown mechanism. To clarify how complement contributes to the early, innate and the late, specific immune response and resulting protection, this study addresses the amount of C3, the timing when its presence is required as well as the anaphylatoxin receptor(s) mediating its effects and the complement-dependent migration of dendritic cells. Challenge experiments with *C. psittaci* on various complement KO mice were combined with transient decomplementation by pharmacological treatment, as well as the analysis of *in vivo* dendritic cells migration. Our findings reveal that a plasma concentration of C3 close to wildtype levels was required to achieve full protection. The diminished levels of C3 of heterozygote C3^+/−^ mice permitted already relative effective protection and improved survival as compared to C3^−/−^ mice, but overall recovery of these animals was delayed. Complement was in particular required during the first days of infection. However, additionally, it seems to support protection at later stages. Migration of CD103^+^ dendritic cells from the infected lung to the draining lymph node—as prerequisite of antigen presentation—depended on C3 and C3aR and/or C5aR. Our results provide unique mechanistic insight in various aspects of complement-dependent immune responses under almost identical, rather physiological experimental conditions. Our study contributes to an improved understanding of the role of complement, and C3a in particular, in infections by intracellular bacteria.

## Introduction

Chlamydiae are gram-negative bacteria with a unique productive cycle. As metabolically (almost) inactive elementary bodies (EBs) they induce their own uptake into mucosal cells. Escaping endolysosomal degradation after infection, they remain in intracellular inclusions as metabolically active, dividing reticulate bodies. Chlamydiae modify the host cell by translocation of effector proteins via their type III secretion system and diminish the immune response. Finally, a new generation of infectious EBs is released by extrusion or cell lysis [as reviewed elsewhere (1)]. Within a broad host spectrum depending on species and serovar, Chlamydiae cause diseases in mucosal organs (2, 3). In humans, *Chlamydia trachomatis* is the main cause of bacterial urogenital-infection with infertility as one sequel (4). Moreover, serovars A-C can cause trachoma and blindness (5). *Chlamydia pneumoniae* leads to respiratory infections (6).

Zoonotic *Chlamydia psittaci* (*C.ps*.) causes intestinal, ocular and respiratory infections in birds. These BSL 3 strains can be transmitted to humans. As a result, patients can develop atypical pneumonia with systemic spread of the pathogen (7) and mortality rates of up to 20% (8). The number of reported cases in economically developed countries is generally low, partially due to preventive measures in poultry and bird farms, and partially due to the absence of *C.ps*. in most routine diagnostic schemes. Yet, in some regions of Germany up to 2% of cases of community-acquired pneumonia seem to be caused by this pathogen (9, 10). Infections of domestic animals with the closely related non-avian strains, such as the DC-15 isolate from a calf abort used in this study, which are considered BSL 2 by German governmental authorities, are frequent and result in abortion, respiratory disorders, enteritis, arthritis, or protracted animal development and economic loss (11).

Our understanding of the immunological mechanisms shaping chlamydial diseases is limited. Chlamydial infections are often long-lasting and immunopathology influences pathogenesis. Reinfections are possible and development of protective vaccines is difficult [for a review see (12)]. Complement plays an essential role in the defense against *Chlamydia*. Yet, the role of complement-mediated immunity directed against intracellular pathogens, in particular against bacteria that propagate in this confined space is rather unclear.

Complement is a complex, early-activated component of the immune system that cross-links innate and adaptive immunity. It plays an essential role in the defense against pathogens. It consists of >50 serum proteins, mediators, regulators, and receptors. Complement factors are mainly secreted (usually in their inactive form) by hepatocytes, but they are also produced in epithelial, endothelial and adipose tissue (13, 14), or locally by immune cells including dendritic cells (DCs) (14).

In the complement cascade, the majority of effector functions is downstream of and depending on complement factor 3 (C3). The three main activation pathways converge in the formation of a C3 convertase, which cleaves inactive C3 into biologically active C3a and C3b. The anaphylatoxins C3a and C5a (the soluble cleavage product of C5 further downstream in the cascade) activate leucocytes and important inflammatory as well as immunoregulatory pathways via binding to their specific receptors (C3aR, C5aR) (15). Moreover, they can regulate homeostasis and survival of resting T-cells, and human T-helper type 1 (TH_1_) responses (16–19). It is controversially discussed how far these mechanisms also apply to the murine system: There are studies suggesting lymphoid expression of anaphylatoxin receptors. However, there are also opposite findings, e.g., on a naïve anaphylatoxin receptor knock-in reporter mouse expression on lymphocytes could not be demonstrated (20–23). Nevertheless, APC-T-cell (co)-stimulation (20, 24) and development of specific CD4^+^ and CD8^+^ responses against several bacteria and viruses depend also on C3a (25–27). In addition, complement promotes DC migration independent of inflammatory stimuli (e.g., LPS) during *in vivo* Influenza infection, as it is hampered in C3^−/−^ mice (28). Covalent deposition of newly generated and highly reactive C3b on target surfaces opsonizes pathogens and apoptotic cargo and enhances B-cell activation and antibody class switch (29–31). Surface-bound C5b promotes the assembly of the membrane attack complex (13, 32, 33).

During infections, complement plays a key role in immune modulation. It is highly active against extracellular bacteria and viruses, and local production of complement by myeloid-derived cells is believed to be critical (15, 34, 35). Although the concept of C3-dependent effector molecules in controlling immune functions is well-established in the field, we are only beginning to understand their full potential during infections by intracellular bacteria, e.g., Chlamydiae. C3 activation products bind covalently to the surface of EBs of *C. trachomatis*. Moreover, *in vitro* serum neutralization of Chlamydia depends on complement (36, 37). Opsonization of *C. trachomatis* by C3b facilitated rapid uptake in human monocytes (38). Finally, complement was superior to antibodies to facilitate opsonophagocytosis of *C. pneumoniae in vitro* (39).

We have previously demonstrated in a mouse lung infection model that C3 and C3a with its receptor are important for an effective defense against *C.ps. in vivo*: Activation of complement occurs already during the first days, and C3 seems to stimulate via C3a protective, adaptive, cellular immunity (40, 41). Yet, so far, the associated mechanisms that are required for the development of protection against *C.ps*. during mouse lung infection were unknown.

Long-term survival of intracellular bacteria depends on the relative well-being of the host. Hence, pathogens with an obligate intracellular life-style, such as the non-avian strains of *C.ps*., had to adapt during evolution by avoiding a too aggressive strategy. Therefore, chlamydial infections tend to be subacute or chronic, which leads to a disease of longer duration. This opens up the possibility to observe in detail biological effects of the various players of host defense on the development of the specific immune response and protection. In this case, these players are C3 and its biologically active cleavage products.

In this study, we elucidated in detail the mechanism how complement C3 and C3a/C3aR augment protection and immune responses against intracellular *C.ps*. in mouse lung infection. For that purpose, challenge experiments with *C.ps*. on C3^+/+^ (WT), C3^+/−^, C3^−/−^, as well as C3aR^−/−^, C5aR1^−/−^, and C3aR^−/−^ × C5aR1^−/−^ mice were combined with transient pharmacological decomplementation, as well as the analysis of *in vivo* DC migration from the infected lung to the draining lymph nodes.

## Materials and Methods

### Chlamydial Culture

The DC-15 strain of *C.ps*. isolated from bovine abortion (42) was kindly provided by K. Sachse (NCBI GenBank accession: CP002806.1) and propagated in BHK-21 cells as described elsewhere (41). The German government and corresponding authorities regard “non-avian *C.ps*. strains” BSL 2 (http://www.zkbs-online.de). Inclusion-forming units (IFU) were assessed by titration using HeLa-T cells (43). Mock infected controls were prepared identically, but without Chlamydia, and diluted to the same extent as harvested infected cells. All Preparations were Mycoplasma-free tested by PCR.

### Animal Experiments and Mouse Strains

All animal experiments were approved by the Lower Saxony state government and corresponding authorities of the Lower Saxony State Office for Consumer Protection and Food Safety (LAVES) (permit: 33.12-42502-04-17/2666, 33.12-42502-04-14/1578, 33.19-42502-04-18/2979, 33.12-42502-04-19/3078). Experiments were performed in accordance with the law of animal welfare used for experiments (TierSchVersV), with the German regulations of GV-SOLAS for the protection of animal life and FELASA. In this work, the following strains were used: C3^−/−^ [B6.129S4-C3tm1Crr/J– (44)], C3^+/−^, C3aR^−/−^ × C3aR^−/−^ [B6.129X1-C3ar1tm1Raw (45) × C5ar1tm1Cge (46)], as well as WT C57BL/6J [JAX-ID 002014] mice. All C3^−/−^, C3aR^−/−^ × C5aR1^−/−^ and mice were backcrossed on C57BL/6J background for 6 additional generations. All single KO strains as well as all cross-breedings were generated from these strains or with the corresponding WT strain.

### *Chlamydia psittaci* Mouse Lung Infection

Intranasal infection of 9–10 week old male mice was performed under anesthesia using 100 mg/kg BW Anesketin (Dechra, 08714225156146), 4 mg/kg Rompun (Bayer, PZN-01320422) in 0.9% NaCl as described elsewhere (47). Depending on the setting, mice were infected with an IFU of 2 × 10^3^ or 4 × 10^4^ per mouse. All infected animals were observed daily assessing clinical score and body weight. For Mock infection (Mock) mice were treated identically, applying the same volume of mock material obtained from cultured non-infected BHK-21 cells (see above in “Chlamydial culture”). All infected animals were observed daily assessing clinical score (for details see Table in Supplementary Material) and body weight.

### Lung Histopathology

Classification of lung histopathology by light microscopy on Hematoxylin and Eosin (Merck) stained deparaffinized, “blinded” lung sections was performed as previously described (41).

### Cryo-Conservation of Lung Tissue and Preparation of Single Cells Suspensions From the Lung Draining Lymph Nodes

Organs from infected and mock treated dissected mice and lung homogenate were cryo-conserved as previously described (40, 41). To obtain single cell suspensions, lymph nodes (LN) were collected in PBS+10% FCS passed through a 40 μM nylon mesh (BD, 352340) using a syringe stamp and flushed two times with 10 ml of cold PBS. If necessary, red blood cell lysis was performed using ACK lysis buffer (155 mM NH_4_Cl, 10 mM KHCO_3_, 0.1 mM EDTA).

### Transient Decomplementation of Mice Using Cobra Venom Factor (CVF)

Complement depletion of mice was performed by intraperitoneal injection of 30 μg (16.2IU) CVF (Quidel, A600, Lot#:120878) in 200 μl PBS to mice at indicated time points. C3 cleavage in mouse plasma was controlled by C3 Sandwich ELISA. Blood of mice was collected at various time points by puncture of the tail vein using EDTA coated capillaries (Sarstedt, 19.447). Collected mouse blood was diluted 1:10 in 10 mM EDTA PBS at a pH of 7.4 to stop further C3 degradation (48). Blood samples were centrifuged for 1 min at 4°C at 500 × g and the supernatant stored at −80°C for analysis.

### Mouse C3 ELISA

Plasma samples from heart puncture (41) or plasma diluted 1:10 as described above in PBS-EDTA were used to perform ELISAs. The capture antibody directed to C3 (Hycult, HM1065 1 μg/ml) was incubated for 16 h at 4°C with Nunc-Immuno™ MicroWell™ 96 well polystyrene plates. Blocking was performed 1 h with 1% BSA and 5% sucrose at RT, followed by three washing steps. Samples were thawed on ice, diluted in reagent dilution buffer (20 mM Tris, 150 mM NaCl, 0.1% BSA and 0.05% Tween-20) and incubated for 1.5 h 37°C on the ELISA plate, followed by washing steps. Plates were incubated with detection antibody in dilution buffer for 45 min at 37°C (0.5 μg/ml biotin rat anti-mouse C3a, Hycult, HM1072-B). After incubation and washing, 10 μg/ml streptavidin-conjugated HRP (Jackson Immuno Research, 016-030-084) was added for 20 min at RT. After washing, substrate buffer (90 mM Na-acetate, 90 mM citric acid, 100 μg/ml TMB, 0.0045% H_2_O_2_) was added to each well and incubated for 20 min in the dark and enzymatic reaction stopped by adding 1M H_2_SO_4_ to each well. Photometric absorbance was measured by Synergy HTC Multi-Mode Reader Biotec^®^ plate reader at 450/540 nm. C3 concentration was calculated by a corresponding standard curve consisting out of pooled C57-BL/6J mouse plasma pre-diluted 1:3 in PBS (10 mM EDTA pH 7.4). Sample analysis was performed by calculating the reversing function of the sigmoidal standard curve using the four parametric logistic equation in Graphpad Prism. In some cases, normalization of data sets was performed to remove technical artifacts and variations between experiments.

### TNF-α, IFN-γ and Myeloperoxidase (MPO) ELISA

These ELISAs were performed in similar fashion as described above according to the manufacturer's protocol (MPO: MPO, Mouse, ELISA kit, Hycult Biotech, HK210-02; IFN-γ: ELISA MAX™ Deluxe Set Mouse IFN-γ, BioLegend, 430804; TFN-α: ELISA MAX™ Deluxe Set Mouse TFN-α, BioLegend, 430904).

### Flow Cytometric Determination of Bacterial Load in Lung Homogenate

1.6 × 10^4^ HeLa-T cells were seeded into a 96 well and cultured in MEM-media 24 h prior to infection. On the next day, cells were washed once with PBS and media was changed to RPMI-1640 media containing 10% FCS. Cryopreserved lung homogenates were thawed 15 min on ice, samples were vortexed for 3 min and centrifuged for 15 min, 500 × g, 4°C. The collected sample supernatant was serial diluted in RPMI 1640, 10% FCS and used to infect HeLa cells. In addition, a dilution series of Chlamydia stock preparation was used as internal standard to calculate bacterial content in lung homogenate samples. Next, cells were spinoculated with diluted supernatant or *C.ps*. stock dilution for 1 h, 2,000 × g, 35°C. 20 h after infection, cells were washed with PBS and trypsinated with Trypsin/EDTA for 5 min. RPMI 1640 10% FCS was added to stop enzymatic digestion and cells were transferred to a v-shaped 96 well plate suitable for staining procedure. Next, cells were spinned down for 5 min, 1,200 × g, 4°C, supernatant was removed and cells were resuspended in PBS containing 2% formaldehyde to fix cells for intracellular staining. After fixation cells were centrifuged (5 min, 1,200 × g, 4°C), supernatant was removed and washed with PBA-S (PBS supplemented with 0.25% BSA, 0.02% sodium azide, 2 mM EDTA, 0.5% Saponin) to permeabilize cells. Staining of intracellular chlamydia and cells was performed for 40 min in PBA-S at 4°C using Pathfinder^®^ containing Fluorescein-conjugated murine monoclonal antibody to chlamydia LPS and 0.1% Evans Blue (0.54 μl per well; Bio-Rad). After staining cells were washed with PBA-S, centrifuged (5 min 1,200 × g, 4°C) and resuspended in PBA for flow cytometric data acquisition. The dotted horizontal line in graphs depicting “Chlamydia per Lung” indicates the limit of detection (LOD) due to unspecific background fluorescence caused by artifacts or cell debris. It is based on the highest result obtained with several non-infected Mock controls, and was not subtracted in order to get even in negative controls calculated very low, hypothetical chlamydial counts. This procedure simplified statistical comparison of samples containing only few chlamydia (or even none) with those containing higher amounts.

### Staining of Lung Cells With Carboxyfluorescein Succinimidyl Ester (CFSE)

CFDA-SE was dissolved in DMSO and stored at −80°C. The working solution of 0.05 mg/ml CFDA-SE, 0.05% DMSO (Sigma-Aldrich, 67-68-5) dissolved in Iscove's Modified Dulbecco's Medium (IMDM, Invitrogen) was prepared shortly before i.n. application. 24 h before organ harvest, mice were anesthetized and 30 μl of the working solution were slowly dropped on top of the nostril considering the breathing rhythm of the animal.

### Lung Tissue Digestion and Enrichment of CD11c+ Cells by Magnetic Beads

Lungs from euthanized mice were dissected and perfused with HBSS-Mg^2+^/Ca^2+^-free containing 1 mM EDTA and collected in cold HBSS+5% FCS to be later cut into small pieces using a razor blade. Grounded tissue was digested with 5 ml per lung of collagenase digestion solution (1 mg/ml collagenase A, Roche, 9001-12-1; one scraper tip DNase1 per mix, AppliChem, 9003-98-9 in HBSS-Mg^2+^/Ca^2+^-free) using gentleMACS C tube (Miltenyi Biotec, 130-093-237) and the GentleMACS TM Octo Dissociator running the 37C_m_LDK_1 program. Samples were centrifuged at 335 × g for 10 min at 4°C RBC and lysis was performed using ACK lysis buffer for 1 min at RT. Lysis was stopped by adding PBS, followed by centrifugation (10 min, 335 × g, 4°C). Cells were resuspended in 5 ml PBE (PBS containing 0.5% FCS, 2 mM EDTA), passed over a 100 μM nylon mesh into a new FalconTM tube and subsequently washed with 5 ml of PBE. The cells were either used for cell analysis by flow cytometry or magnetic separation of cells was performed. Therefore, a total of 1 × 10^7^ cells per organ were centrifuged and resuspended in 400 μl PBE containing 5 μg of CD16 and CD32 antibody mix (BioLegend, 101301) for 10 min on ice. Next, 10 μl of CD11c MicroBeads UltraPure (Myltenyi, 130-108-338) was added to the mix and incubated for 20 min at 4°C. Magnetic separation was performed for 15 min at 4°C. Subsequently, unbound cells were removed and samples washed with PBE to be placed once more on top of the magnet for 10 min at 4°C. Finally, after discarding the supernatant, the cells were used for flow cytometric analysis.

### Cell Analysis in Lung or Lung Draining Lymph Nodes

Immune cell phenotyping of the lung dLNs or cells from magnetic bead purification was performed using the following antibodies: Pacific Blue™ anti-mouse CD11c (BioLegend, 117322), PE anti-mouse CD103 antibody (BioLegend, 121406PE/Cy7 anti-mouse CD11b (BioLegend, 101216), APC anti-mouse I-A/I-E antibody (BioLegend, 107614), APC/Cy7 anti-mouse CD3ε (BioLegend, 100330), APC-Vio770 anti-mouse CD19 (Miltenyi Biotec,130-112-038), APC-Vio770 anti-mouse CD45R (B220) (Miltenyi Biotec, 130-110-849). For most antibodies, the corresponding isotype controls were used. Flow cytometry acquisition was performed with a MACSQUANT Analyzer 10 (Miltenyi Biotec) and data analyzed with FlowJo v.10.4.2 (Becton, Dickinson and Company (BD).

### Calculations and Statistics

GraphPad Prism V8.0.1 (GraphPad Software Inc.) was used for graphical display and statistical evaluation. Usually, infected and mock-treated animals were compared first using an unpaired *t*-test. If significant *C.ps*.-dependent differences were found further statistical analyses were applied. Normal distribution of parametric data was checked for each data set. In the majority of cases logarithmic transformation of data was performed in order to accomplish Gaussian distribution. Parametric data were analyzed either by unpaired *t*-test (2 groups, 1 condition), One-way (2 ≤ groups, 1 parameter) or Two-way ANOVA (2 ≤ groups, 2 ≤ parameter; repeated measurements with mixed effects model for body-weight and C3 plasma quantification after CoVF treatment) followed by Bonferroni multiple comparison test. In general mean ± SD (standard deviation) was used to represent parametric data. In some cases, if the sample size exceeded n ≥ 6, mean ±SEM (standard error of mean) was used. Non-parametric data were shown as mean ± IQR (interquartile range) and analyzed by Kruskal-Wallis test followed by Dunn's multiple comparison post-test. Details and group size of each experiment is depicted within or under each corresponding figure. Survival of mice was analyzed by Mantel-Cox log-rank test. Statistical significance was displayed and presented according to the calculated *p*-value: ns *p* > 0.1234; *p ≤ 0.0332; **p ≤ 0.0021; ***p ≤ 0.0002; ****p ≤ 0.0001.

## Results

### Protection Against *C. psittaci* Depends on the Level of C3

**Figure 1:**
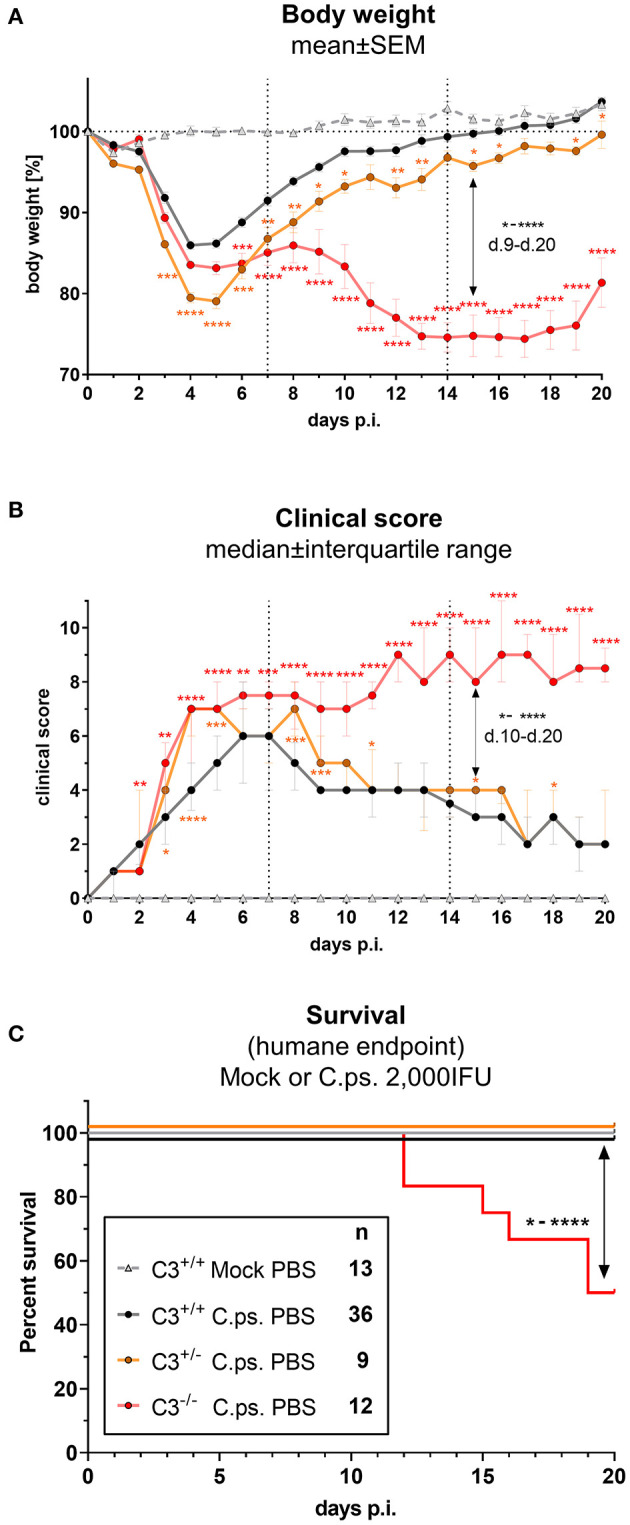
C3 WT-levels are required to achieve optimal protection in *C. psittaci* mouse lung infection, but by 40%-reduced levels are sufficient to sustain critical functions. C3^+/+^, C3^+/−^, and C3^−/−^ mice were i.n. infected with 2,000 IFU of *C.ps* or Mock material. Body weight **(A)** and clinical score **(B)** were determined daily. Under close monitoring and using humane endpoint defining criteria survival was additionally assessed until the intended end of the experiment on day 20 p.i. **(C)**. C3^+/−^ mice showed a reduction of ~40% in basal C3 plasmas level (Supplementary Figure 1); in C3^−/−^ mice no C3 could be detected (data not shown). The depicted results were combined from 3 independent identically performed experiments. Statistical analysis of the data depicted in **(A,B)** was performed by Two-way ANOVA, repeated measurements with mixed effects model followed by Bonferroni post-test, or by Kruskal-Wallis test followed by Dunn's multiple comparison post-test. Statistical analysis of the data concerning the humane endpoint **(C)** was performed by log-rank Mantel-cox test. Statistical significance according to the calculated p-value: *p ≤ 0.0332; **p ≤ 0.0021; ***p ≤ 0.0002; ****p ≤ 0.0001.

In order to elucidate how much C3 is required to achieve full protection against *C.ps*., the course of induced pneumonia in terms of body weight and clinical score was not only investigated in homozygous C3^+/+^ (WT) and C3^−/−^, but also in heterozygous C3^+/−^ mice (Figures 1A–C).

C3 levels of these mice were determined by an ELISA. The C3 level found in EDTA-plasma from pooled WT mice served as standard defining 100 U. The basal C3 levels of C3^+/−^ mice were ~40% lower than those from C3^+/+^ animals (Supplementary Figure 1). As expected, no C3 could be detected in C3^−/−^ animals (data not shown).

Around day 3 and 4 after *C.ps*. infection, the heterozygote mice started to lose more weight and showed a higher clinical score compared to C3^+/+^ animals, and, considering the weight gain, their complete recovery within ~3 weeks was shifted and delayed by 1 to 2 days (Figures 1A,B). These data indicate that an amount of C3 close to WT levels is required to achieve rapidly full protection.

Yet, in animals without any C3, a difference in disease progression with a much more severe second phase of the illness occurred from day 9 to 20 (Figures 1A,B), a period of infection when the specific immune response plays a major role. In accordance with that, roughly half of the C3^−/−^ animals reached the humane endpoint prematurely in the second half of the observation period, whereas all *C.ps*.-infected C3^+/+^ and C3^+/−^ mice survived 20 days (Figure 1C). That demonstrates that the residual C3 in the heterozygote mice was sufficient to maintain crucial functions of defense.

### Optimal Defense Against *C. psittaci* Infection Depends on Early Complement Activation

CVF purified from the venom of the spectacled cobra forms in complex with complement factor B and factor D a stable C3 convertase. That leads to nearly complete cleavage of C3 and C5, thus causing rapid transient consumption of complement *in vivo* without any symptoms of disease (49, 50). Hence, application of CVF to mice can be used to obtain insight into the period, when presence of complement is required to induce (via its activation products) protection during different stages of *C.ps*. infection.

**Figure 2:**
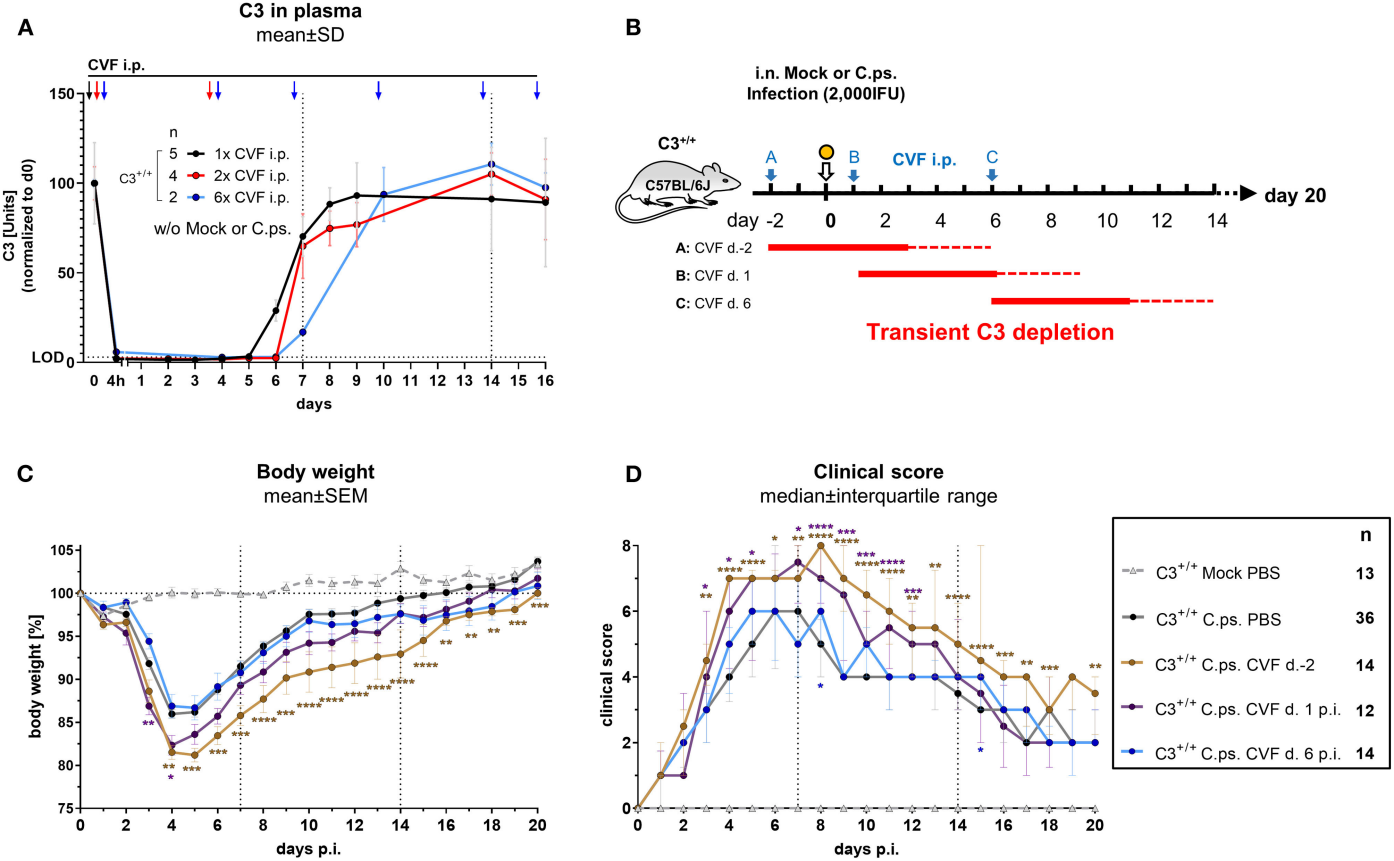
Presence of C3 during the first days of *C. psittaci* lung infection is required to provide optimal protection. Healthy, non-infected C3^+/+^ mice were treated 1, 2, or 6 times i.p. with Cobra venom factor (CVF) as indicated by the black, red, or blue arrows, respectively. EDTA plasma from these mice was collected at the indicated time-points, and C3 levels were determined by ELISA. Pooled plasma from WT mice served as standard defining 100 U **(A)**. To assess the relevance of complement at different phases of *C.ps*. infection, C3 was transiently depleted by single application of CVF 2 days before, or on day 1 or 6 after *C.ps*. (2,000 IFU) infection. In this scheme, the blue arrows indicate the different time-point of CVF application, red horizontal lines represent the expected resulting periods of decomplementation **(B)**. Infected and mock-infected control mice were treated with PBS instead of CVF (dark circles and light gray rectangle in **C,D**). Body weight **(C)** and clinical score **(D)** were assessed daily. The results of Mock-infected C3^+/+^ mice receiving PBS instead of CVF are also depicted in these graphs. Of all mice, only 2 of 12 on day 2 pre-infection CVF-treated mice reached the humane endpoint prematurely (data not shown). The results depicted in **(A,C,D)** were combined from four, independent experiments. Statistical analysis was performed by Two-way ANOVA (**A,C**), repeated measurements with mixed effects model followed by Bonferroni post-test, or by Kruskal-Wallis test followed by Dunn's multiple comparison post-test **(D)**. Statistical significance according to the calculated *p*-value: *p ≤ 0.0332; **p ≤ 0.0021; ***p ≤ 0.0002; ****p ≤ 0.0001. Illustrations were partially created using templates from www.motifolio.com. d., day; LOD, limit of detection.

A single i.p. CVF application in non-infected animals led to a 4 to 5 days lasting phase with C3 plasma levels below the detection limit of the C3-ELISA used (black arrow, black line and dotted horizontal line marked with LOD in Figure 2A). This phase was prolonged only for ~24 h when CVF was administered a second time on day 3 (red arrows and line in Figure 2A). Repetitive application of CVF on day 0, 3, 6, 9, 13, and 15 (as indicated by the blue arrows and line in Figure 2A) was almost without any further effect on the reappearance of C3 in plasma.

**Figure 3:**
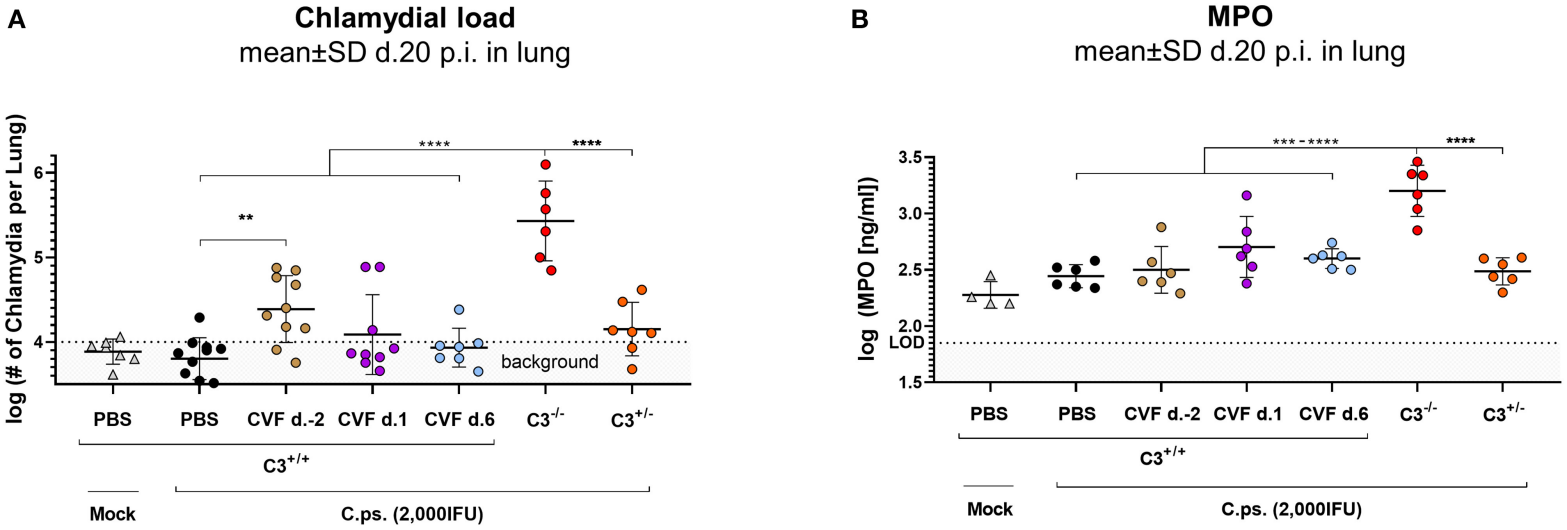
WT-C3 levels of C3^+/+^ mice and presence of this central complement factor (for activation) during the first days of lung infection are required to provide optimal clearance of *C. psittaci*. The chlamydial load **(A)** and the level of the granulocyte marker MPO **(B)** were determined in the lung of mice 20 days p.i with 2,000 IFU of *C.ps*. or Mock material. Their complement was either transiently depleted by single application of CVF to C3^+/+^, i.e., WT animals, as indicated (2 days before, or on day 1 or 6 after infection), or it showed different basal levels of C3 according to their genetic status as C3^+/+^, C3^+/−^, and C3^−/−^ C57BL/6J mice. The depicted results were combined from three or more, independent identical experiments. Statistical analysis was performed by One-way ANOVA followed by Bonferroni post-test. Statistical significance was displayed according to the calculated *p*-value: *p ≤ 0.0332; **p ≤ 0.0021; ***p ≤ 0.0002; ****p ≤ 0.0001. d., day; LOD, limit of detection.

Hence, all subsequent transient CVF experiments on *C.ps*. infected mice (2,000 IFU per mouse) were performed using a single dose administered either on day −2, or +1, or +6, respectively (Figure 2B with scheme of estimated times without circulating C3 as red horizontal lines, and the corresponding results in Figures 2C,D). The largest effect of transient decomplementation compared to PBS-treated WT mice was achieved when complement was absent between day −2 and ~2 to 3 of infection (brown line in Figures 2C,D): The loss of body weight was more severe, recovery delayed by at least 3 days and the chlamydial load in lung homogenate remained elevated on day 20 p.i (Figure 3A). The effect was smaller when CFV was applied 1 day after infection (and circulating C3 is expected to lack until day 5 or 6—violet line in Figures 2C,D). There was no visible effect when CFV was applied on day 6 p.i. (with an estimated time without circulating C3 until day 11). Corresponding to that, in contrast to all other groups ~15% of infected mice from the on day −2 CFV-treated group reached the humane endpoint prematurely beginning at day 14 (brown line in Supplementary Figure 2).

Most likely due to emerging neutralizing antibodies against CVF (51), a more permanent pharmacological consumption of complement by repetitive application of this snake derived protein, mimicking the C3^−/−^ mouse, could not be achieved. Thus, with this type of experiment alone we could neither proof nor exclude that complement must be present for more than the first 4 to 5 days to achieve full protection.

Taken together, our data indicate that C3-dependent processes during the first days of infection increase protection against *C.ps*.

### Complement Depletion and Absence of C3 Early in Infection Impairs Bacterial Clearance and Increases Neutrophil Infiltration (Indicated by MPO) in the Infected Lung

In order to further assess the importance of the level of C3 and the period of its presence for protection in *C.ps*. lung infection, bacterial clearance from the lung and the granulocyte-marker MPO were additionally determined in the CVF-treated and transiently decomplementated as well as the C3^+/+^ (WT), C3^+/−^, and C3^−/−^ mice (Figures 3A,B).

Concluding from the chlamydial load in the lung, chlamydial clearance was slightly impaired when complement was absent due to CVF-treatment between day −2 and ~2 to 3. Moreover, similar to the C3^+/+^ and in contrast to C3^−/−^ mice, the heterozygous mice were able to eliminate to a great extend the intracellular bacteria until day 20 (Figure 3A). At that time point, MPO in the lung homogenate was highly elevated only in C3^−/−^ animals (Figure 3B).

Taken together, these results underline the relevance of complement early in *C.ps*. infection. However, comparison of the transiently decomplementated with the much more severe phenotype of the C3^−/−^ mice (Figures 3A,B) also strongly suggests that presence of complement during the first few days p.i., although critical, is (alone) not sufficient to achieve full protection.

### Anaphylatoxin C3aR, but Not C5aR1, Mediates Protection in the Defense Against *C. psittaci*

**Figure 4:**
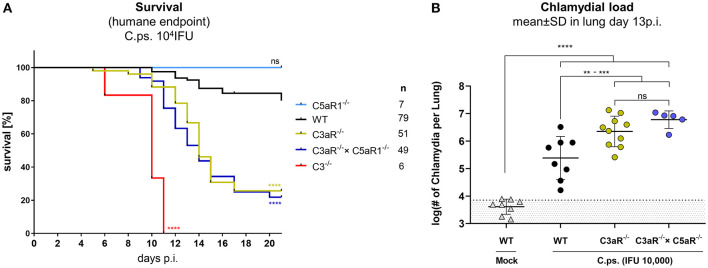
C3a receptor (C3aR) but not C5a receptor 1 (C5aR1) is protective against *C. psittaci*. WT, C3^−/−^ and C3aR^−/−^ and/or C5aR1^−/−^ mice were challenged with 10^4^ IFU of Chlamydia. Under close monitoring and using humane endpoint defining criteria survival was assessed **(A)**. To permit comparison, the chlamydial burden in the lung (of additional mice) was determined on day 13 p.i **(B)**. Statistical analysis was performed by log-rank Mantel-cox test **(A)** or by One-way ANOVA followed by Bonferroni post-test **(B)**. Statistical significance according to the calculated *p*-value: *p ≤ 0.0332; **p ≤ 0.0021; ***p ≤ 0.0002; ****p ≤ 0.0001.

The anaphylatoxic peptides C3a and C5a can modulate immune responses and inflammation. *C.ps*. challenge experiments were performed under more stringent conditions (10,000 IFU) in C3aR^−/−^, C5aR1^−/−^, C3aR^−/−^ × C5aR1^−/−^ (the double KO for the first time), C3^−/−^ and WT mice to clarify how far the anaphylatoxins account for the observed complement dependent protection in *C.ps*. lung infection and in particular, whether C3a and C5a cooperate or are functionally redundant (Figures 4A,B). All C3^−/−^ mice reached the humane endpoint already between day 6 and 11 p.i. The Kaplan-Meier plot demonstrates an almost identical, still severe, but less drastic course of the infectious disease for C3aR^−/−^ and C3aR^−/−^ × C5aR1^−/−^ animals. In contrast, all C5aR1^−/−^ and ~80% of the WT mice survived 20 days of *C.ps*. infection (Figure 4A). Obviously, even in absence of the C3aR, C5a, and C5aR1 are not protective—excluding redundancy or synergy of the two related anaphylatoxin receptors.

The C5aR1^−/−^ mouse was therefore not included in further analysis. We know already from former experiments (under slightly different experimental conditions), that, compared to C3^+/+^ mice, there are much higher numbers of chlamydia on day 9 (but not on day 4) in the lungs of C3^−/−^ mice (41). On day 13, ~half of the *C.ps*.-infected C3aR^−/−^ and C3aR^−/−^ × C5aR1^−/−^ mice were still alive (Figure 4A) permitting then determination of the chlamydial load (Figure 4B). The numbers of vital chlamydia in the lung of equally treated KO animals was at that time-point >10-fold higher than in WT animals clearly demonstrating an essential role of the anaphylatoxin C3aR.

### CD103^+^ DC Migration to the Draining Lymph Node Depends on Complement Activation and Anaphylatoxin Receptor Signaling

Migration of antigen loaded DCs from the infected organ to the dLN for antigen-presentation is one prerequisite for the induction of an effective specific T-cell response (52). The outcome of this response depends on the antigen-presenting DC subset, the presented antigen, as well as the cytokine secretion profile of the DCs during the T-cell priming process (53–55). DCs are known to express complement components and receptors including C3aR and C5aR1 (56–58), and C3 secretion by DCs is required for the induction of TH_1_ responses (24). A recent study of Shekhar et al. demonstrates that transferred lung CD103^+^- and CD11b^+^-DCs from *Chlamydia muridarum* infected mice are both capable to induce protection against this chlamydial mouse pathogen, whereas CD103^+^-DC transfer was associated with higher protective capacities and induction of TH_1_ responses (59). Intriguingly, the protective immune response to viruses depends on C3 and C3a/C5a-mediated DC migration (28).

**Figure 5:**
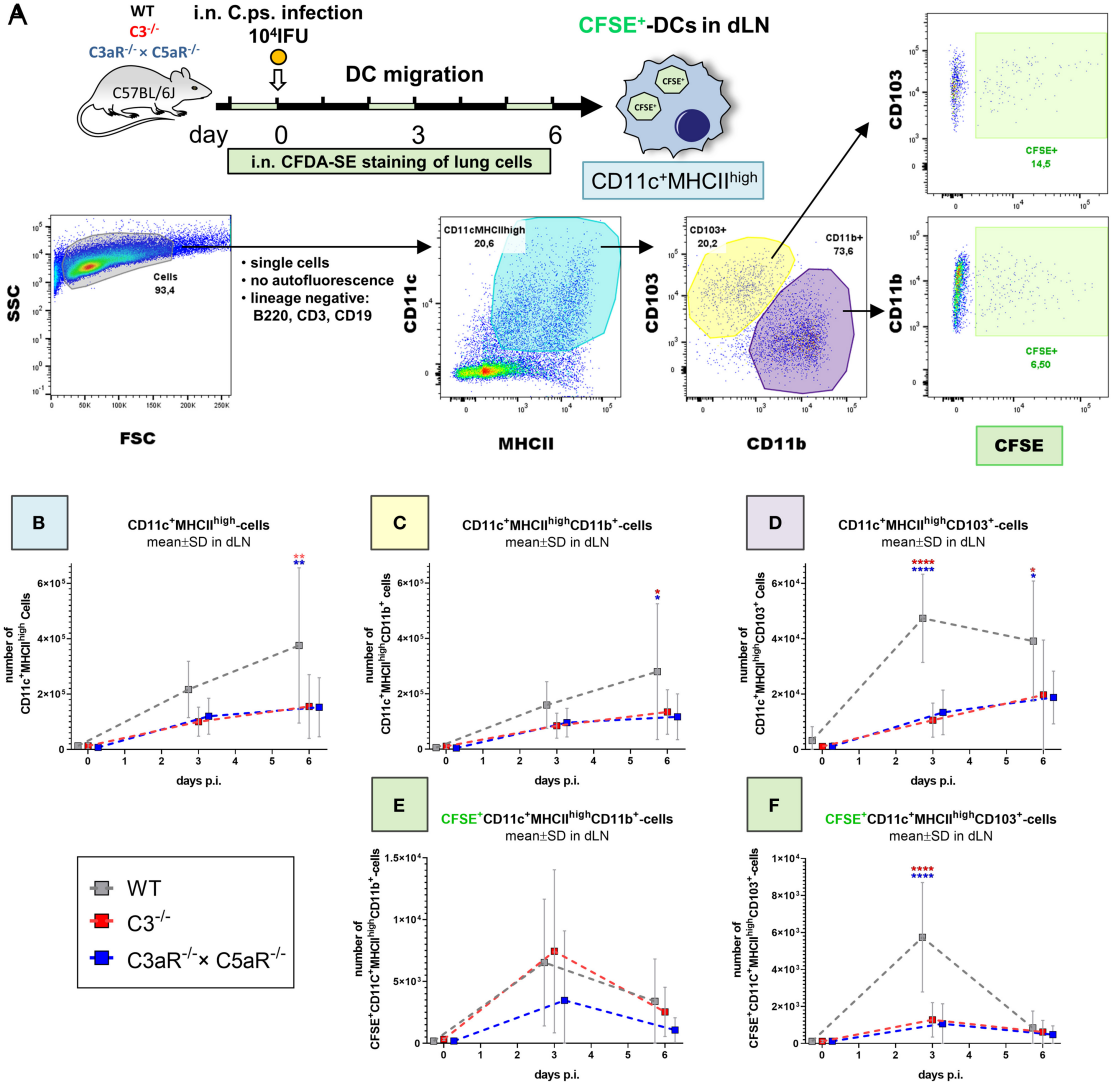
Migration of DCs from the *C. psittaci* infected lung to dLN depends on C3 as well as C3aR (and C5aR1). DC migration to the dLN was assessed on day 0 (i.e., directly before), 3 and 6 of *C.ps*. infection (10^4^ IFU of Chlamydia). Considering isotype and FMO controls and distinguishing between CD103^+^ and CD11b^+^ subsets, DCs were identified as lineage negative non-auto-fluorescent CD11c^+^MHCII^high^-expressing cells. Applying i.n. *in vivo* staining of lung cells 1 day before analysis, lung DCs which had migrated during the last ~24 h could be identified as CFSE^+^ cells in the dLN (as explained by the scheme in **(A)**. The numbers of CD11c^+^MHCII^high^ **(B)**, CD11c^+^MHCII^high^CD11b^+^ **(C)**, and CD11c^+^MHCII^high^CD103^+^ **(D)** DCs in steady state were determined at the indicated time-points p.i. The kinetic of migratory lung CFSE^+^-CD11b^+^- and CD103^+^ DCs in dLN is depicted in the lower two panels **(E,F)**. Statistical analysis was performed by One-way ANOVA followed by Bonferroni post-test. Statistical significance was displayed according to the calculated *p*-value: *p ≤ 0.0332; **p ≤ 0.0021; ***p ≤ 0.0002; ****p ≤ 0.0001. Illustrations were partially created using templates from www.motifolio.com. DC, dendritic cell; FMO, fluorescence minus one control.

Hence, we were interested to clarify how far migration of CD103^+^ and CD11b^+^ DC subsets during the first days of *C.ps*. infection from the infected lung to dLN also depends on C3 and the C3a and C5a anaphylatoxin receptors (Figure 5).

DCs in the lung dLN were defined as non-autofluorescent CD11c^+^MHCII^high^ cells and their subpopulations classified based on the expression of CD103^+^ and CD11b^+^ (Figure 5A). The number of DCs in the dLN of WT animals rose from day 0 (i.e., immediately before infection) to day 3 and 6 p.i. (Figure 5B). Compared to cells from WT mice, this increase of the steady state level was smaller in both, C3^−/−^ and C3aR^−/−^ × C5aR1^−/−^ (double KO) mice. On day 6 it was diminished for CD11c+MHCII^high^ and for CD11c^+^MHCII^high^CD11b^+^ cells (Figures 5B,C), and on day 3 and 6 for CD11c^+^MHCII^high^CD103^+^ DCs (Figure 5D). In order to determine the number of DCs which had actually migrated within the last ~24 h from lung tissue to the dLN, CFDA-SE was applied i.n. one day before cell harvest. Maximal migration of stained DCs occurred on day (2 to) 3 p.i. in WT mice (Figures 5E,F). At this time point, smaller numbers of migrated CFSE^+^CD11c^+^MHCII^high^CD103^+^ cells were present in both, C3^−/−^ and C3aR^−/−^ × C5aR1^−/−^ animals (Figure 5F).

In the *C.ps*.-infected lung of WT and C3^−/−^ and C3aR^−/−^ × C5aR1^−/−^ mice the same DC subpopulations were also determined. Only on day 6 p.i., a relevant increase of DCs with lower numbers of CD11c^+^MHCII^high^ and CD11c^+^MHCII^high^CD11b^+^ cells in the absence of C3 or C3aR and/or C5aR1 in that organ occurred (Supplementary Figures 3A–C).

The results from dLN and lung strongly suggest that the differences observed in the number of DCs in the dLN on day 3 in general, and on day 6 for CD11c^+^MHCII^high^CD103^+^ DCs (Figures 5B–F) directly depend on a complement-related defect in DC migration. Elevated numbers of CD11c^+^MHCII^high^CD11b^+^ DCs in absence of C3, C3aR, and/or C5aR1 in the lung on day 6 p.i might additionally indicate decreased complement-dependent recruitment of this cell type to the infected lung at later stages of infection (Supplementary Figure 3B).

## Discussion

Our results demonstrate that C3 WT-levels are required for maximal protection in *C.ps*. lung infection: A reduction of ~40% of the C3 basal level in C3^+/−^ mice led already to a delay of 1 to 2 days in clinical recovery and in weight gain as compared to infected WT mice. Nevertheless, the remaining C3 in heterozygote mice was sufficient to achieve normal bacterial clearance until day 20 p.i., and the number of granulocytes in the lung (according to MPO as marker) also normalized by then. These observations suggest that complement is required to pass rapidly through the inflammatory phase of infection. The early difference in the course of the lung disease of WT and C3^+/−^ mice and the kinetics from day 5 on point to changes of the early, innate immune response, when C3 is only reduced, but not absent.

In addition, we obtained direct insight into the period, when presence of complement is required (in order to be activated) to induce protection against *C.ps*.. According to the results of the transient decomplementation experiments with CVF, complement is required at the beginning and during the first days of *C.ps*. infection to achieve optimal protection. However, comparison of the results obtained after early decomplementation with the more severe late phenotype of the C3^−/−^ mouse, where C3 is permanently missing, strongly suggests that complement must act at early *and* late stages of infection (most likely on day 6 and later) to induce only in this combination a second phase of a protective, adaptive immune response.

The results obtained with different KO mouse strains exclude a significant role of C5a/C5aR1 in the defense against *C.ps*. and confirm former reports (40, 41) that the C3 cleavage product C3a and its receptor strongly contribute to observed elimination of the intracellular bacteria. However, our data also show that C3aR-signaling alone does not explain all C3-dependent effects.

As discussed above, presence of complement during the early stage of infection is important. Corresponding to that, migration of CD103^+^ DCs from the *C.ps*. infected lung to the dLN depended on C3 and C3aR and/or C5aR1 around day 3, and the increase of DCs in the *C.ps*.-infected lung—again depending on complement—occurred slightly later, around day 6. Similar results have been described in experimental Influenza virus infection (28). Future studies will show how far maturation of DCs might also be affected in the absence of C3 or C3a. However, at least in Influenza infection, maturation of migratory DCs is not dependent on complement C3 or its cleavage products (28). Most likely, increased DC migration (for antigen presentation in the dLN), in particular of the CD103^+^ subset, contributes to the development of the more effective specific immune responses of B- and T-cells and the protection of complement-competent WT mice during the late phase of *C.ps*. infection.

The analysis of the complement-dependent B- and T-cell responses against *C.ps*. is beyond the scope of this project. These aspects have been addressed in parallel, in a closely related project, thereby additionally elucidating the role of non-myeloid- and myeloid-cell derived complement.

The present study provides an improved general understanding of the potential role of complement in human infections with an intracellular bacterium. Furthermore, in regard to complement factors, reports indicating large interindividual variations in man exist, e.g., of the MBL serum levels, which might explain donor-to-donor variations in chlamydial serum susceptibility (60–62). Thus, the different course and outcome of *C.ps*. infections with avian-strains in man might partially depend on the individual levels of C3, or of other factors participating in the activation or effector pathways of the complement cascade. Thus, on the long run, it might be possible to identify patients that are more susceptible and to provide them in *C.ps*. infections with closer monitoring and earlier interventions. It might also be worthwhile to investigate, whether the dangerous avian strains lead to a different degree of complement activation in human serum as compared to the less dangerous non-avian strains. Finally, interindividual differences in the level of complement factors might also influence the course of more chronic infections with *C. trachomatis* or *C. pneumoniae*.

Unfortunately, transfer of complement is presently no therapeutic option. However, the important role of complement that was revealed in the present study might be considered in the design of a vaccine against chlamydia. There are already successive studies based on this thought achieving improved specific immune responses against other pathogens. There, complement activating adjuvants such as Algammulin and gamma-IN (63) or conformational-biased, response-selective agonists of complement components, e.g., of C5a: EP54, or EP67 (64) are used. For that purpose, it might also be worthwhile to generate fusion proteins of chlamydial antigens and the active C-terminal part of C3a as a molecular adjuvant (29).

In summary, our results provide unique insight in various aspects of interacting complement-dependent immune responses under almost identical, rather physiological experimental conditions. Our comprehensive findings on *C.ps*. mouse lung infection comprise (1) the dose dependency of several biological functions on C3, (2) the critical early and, most likely, also late time-point when complement is required, as well as (3) the C3a/C3aR signaling which is partially responsible for protection and (4) complement induced migration of DCs. It is tempting to speculate that our new findings might also apply to infections with other chlamydial species or intracellular pathogens, and even to those with extracellular pathogens causing prolonged infections. Future studies are required to clarify this hypothesis.

## Data Availability Statement

The raw data supporting the conclusions of this article will be made available by the authors, without undue reservation.

## Ethics Statement

The animal study was reviewed and approved by Lower Saxony state government and corresponding authorities of the Lower Saxony State Office for Consumer Protection and Food Safety (LAVES) (permit: 33.12-42502-04-17/2666, 33.12-42502-04-14/1578, 33.19-42502-04-18/2979, 33.12-42502-04-19/3078).

## Author Contributions

MK investigation (performed mouse model and analyses of the obtained samples, in particular flow cytometry), formal analysis (statistics), methodology (established new methods in the lab, e.g., for DC migration), visualization (original graphs), participated in conceptualization and planning, and wrote most of the initial and part of the final draft of the manuscript. CR performed experiments with the samples obtained in the mouse model, e.g., ELISAs. RL performed mouse model and part of the analyses of the samples obtained there. SG performed blinded histological scoring of mouse lung tissue. CL participated in the mouse model. AK conceptualization, funding acquisition, supervision, visualization (optimization of graphs), wrote parts of the initial draft, and optimized the manuscript for submission. All authors contributed to the article and approved the submitted version.

## Conflict of Interest

The authors declare that the research was conducted in the absence of any commercial or financial relationships that could be construed as a potential conflict of interest.

## Supplementary Material

Supplementary Figure 1 Heterozygous C3^+/+^ mice have reduced basal C3 plasma levels. Healthy, i.e., non-infected C3^+/−^ and C3^+/+^ C57BL/6J mice were i.p. treated twice with Cobra venom factor (CVF) as indicated by the two black arrows. EDTA plasma was collected at indicated time-points and C3 levels were determined by ELISA. Pooled plasma from WT mice served as standard defining 100 U. The basal C3 levels of C3^+/−^ mice before and >8 days after CVF application and transient decomplementation were ~40% lower than those from the corresponding WT animals. As expected, no C3 could be detected in C3^−/−^ animals (data not shown). Statistical analysis was performed by TWO-way ANOVA followed), repeated measurements with mixed effects model followed by Bonferroni post-test. Statistical significance was displayed according to the calculated *p*-value: **p* ≤ 0.0332; ***p* ≤ 0.0021; ****p* ≤ 0.0002; *****p* ≤ 0.0001.

Supplementary Figure 2 Survival of CVF-treated C57BL/6J mice during *C. psittaci* lung infection. WT mice were i.n. infected with 2.000 IFU *Cp.s*. Complement was transiently depleted during different phases of infection for ~5 consecutive days (according to the predetermined kinetic) using a single i.p. application of 30 μg CVF (16.2 IU) in PBS. Monitoring closely and using humane endpoint defining criteria survival was assessed. Data were combined from four independent identically performed experiments. Statistical analysis was performed by log-rank Mantel-cox test. Statistical significance according to the calculated *p*-value: **p* ≤ 0.0332; ***p* ≤ 0.0021; ****p* ≤ 0.0002; *****p* ≤ 0.0001.

Supplementary Figure 3 Kinetics of the number of DCs in the mouse lung before and during the first days of *C. psittaci* infection in WT, C3^−/−^ and C3aR^−/−^ × C5aR1^−/−^ mice. Amount of DCs in the lung immediately before and during *C.ps*. infection. Lung CD11c^+^-cells were purified by magnetic bead separation. DCs were identified as lineage-non auto fluorescent CD11c^+^MHCII^high^-expressing cells and subtypes classified based on the expression of CD103^+^ or CD11b^+^, considering isotype and FMO controls. The numbers of CD11c^+^MHCII^high^ **(A)** CD11c^+^MHCII^high^CD11b^+^ **(B)** and CD11c^+^MHCII^high^CD103^+^ **(C)** in the lung were determined at day 0, 3, and 6 post *C.ps*. infection. Statistical analysis was performed by One-way ANOVA followed by Bonferroni post-test (*n* = 5–6 in each group and for each time-point; data were combined from four independent identical experiments). Statistical significance was displayed according to the calculated *p*-value (**p* ≤ 0.0332; ***p* ≤ 0.0021; ****p* ≤ 0.0002; *****p* ≤ 0.0001). *C.ps., Chlamydia psittaci*; DC, dendritic cell; FMO, fluorescence minus one control.

Supplementary Table 1 Clinical scoring.

Supplementary Table 2 Scoring system to evaluate the pathology of deparaffinized lung sections.
