# Supplementary figures and images for: Complement and Chlamydia psittaci: Early Complement-Dependent Events Are Important for DC Migration and Protection During Mouse Lung Infection
Source: Front Immunol. 2021 Mar 9;12:580594. doi: 10.3389/fimmu.2021.580594 (PMC7986412; doi:10.3389/fimmu.2021.580594)

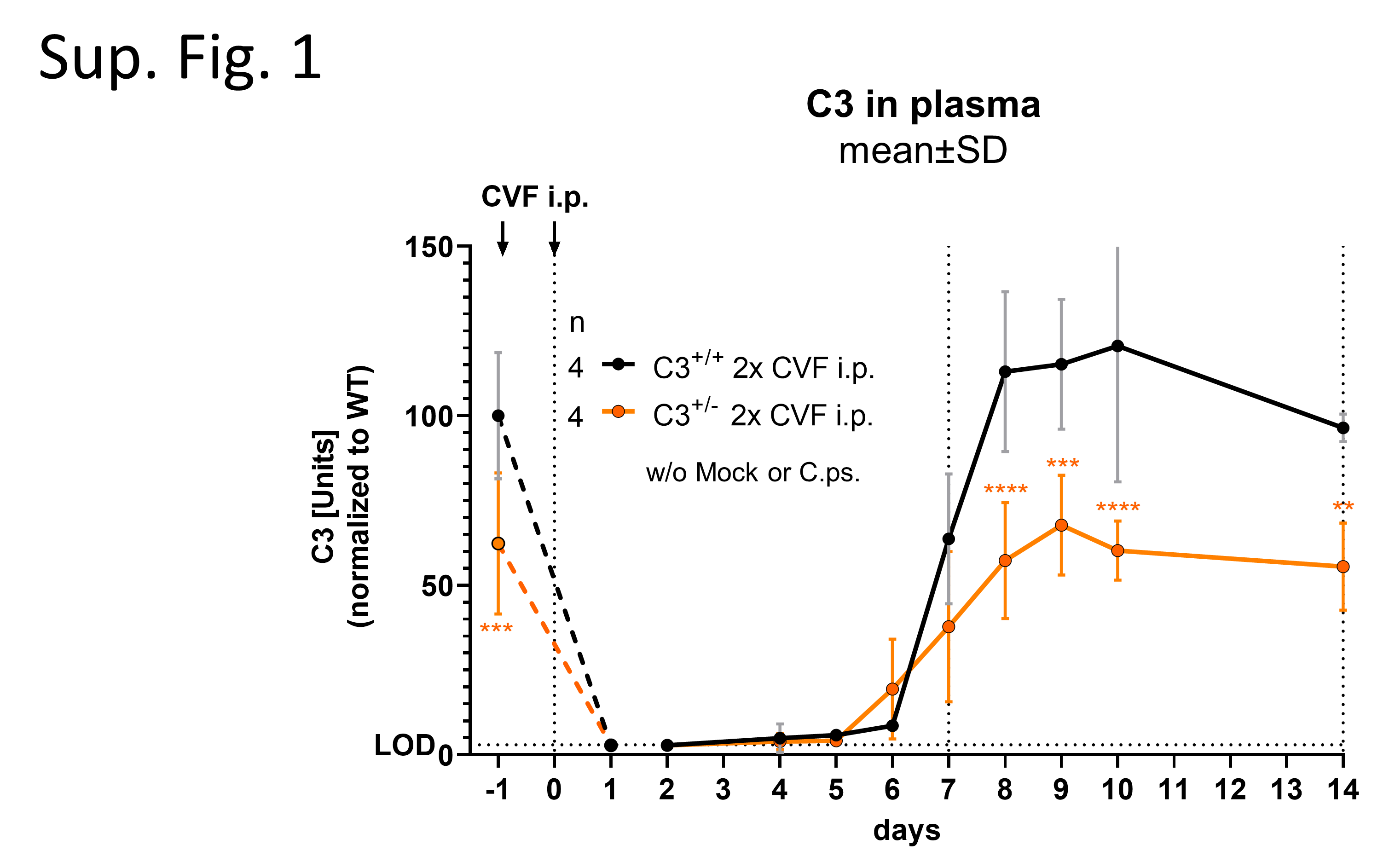

Supplement: Supplementary Figure 1 — Heterozygous C3+/+ mice have reduced basal C3 plasma levels. Healthy, i.e., non-infected C3+/− and C3+/+ C57BL/6J mice were i.p. treated twice with Cobra venom factor (CVF) as indicated by the two black arrows. EDTA plasma was collected at indicated time-points and C3 levels were determined by ELISA. Pooled plasma from WT mice served as standard defining 100 U. The basal C3 levels of C3+/− mice before and >8 days after CVF application and transient decomplementation were ~40% lower than those from the corresponding WT animals. As expected, no C3 could be detected in C3−/− animals (data not shown). Statistical analysis was performed by TWO-way ANOVA followed), repeated measurements with mixed effects model followed by Bonferroni post-test. Statistical significance was displayed according to the calculated p-value: *p ≤ 0.0332; **p ≤ 0.0021; ***p ≤ 0.0002; ****p ≤ 0.0001. [file Image_1.TIF]

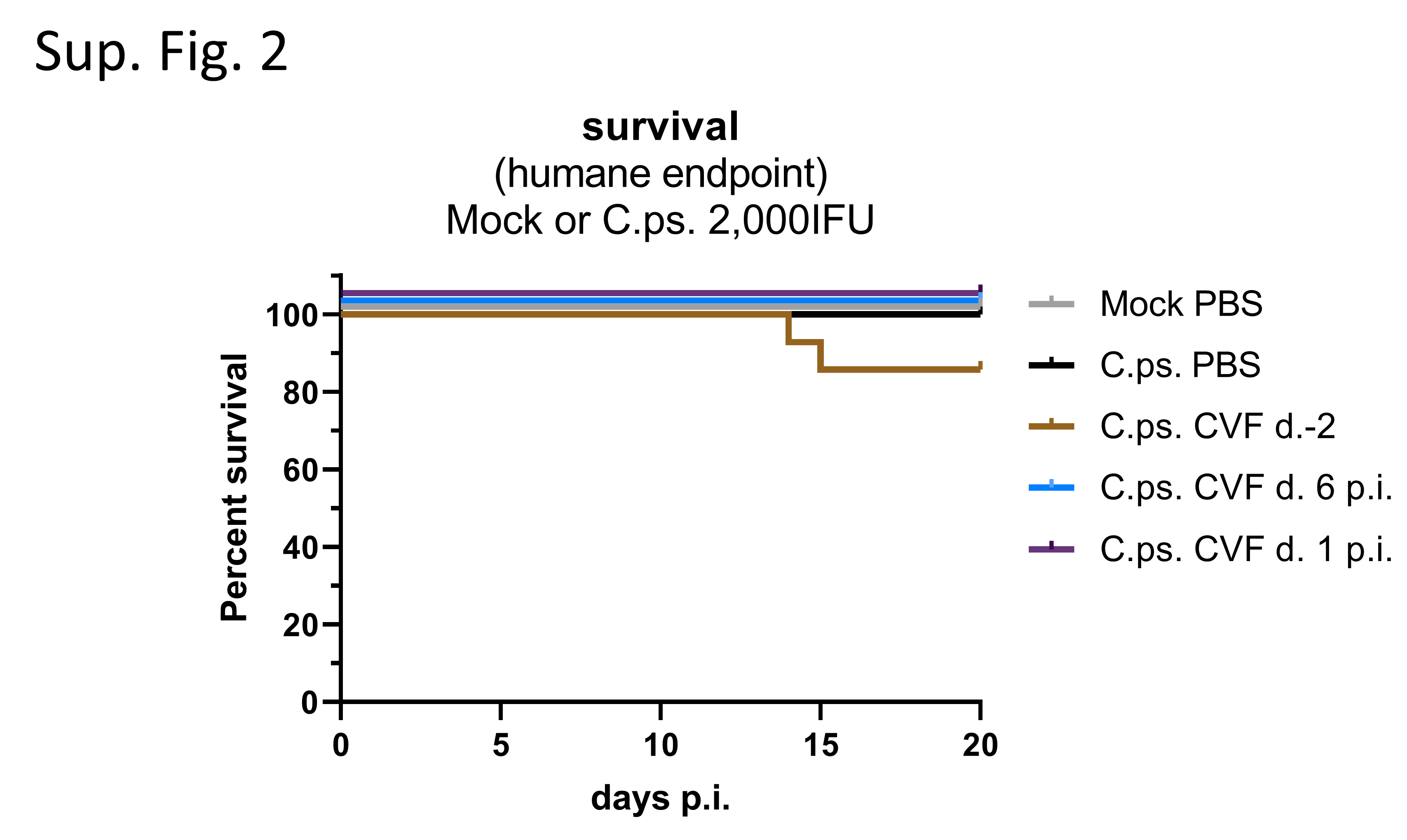

Supplement: Supplementary Figure 2 — Survival of CVF-treated C57BL/6J mice during C. psittaci lung infection. WT mice were i.n. infected with 2.000 IFU Cp.s. Complement was transiently depleted during different phases of infection for ~5 consecutive days (according to the predetermined kinetic) using a single i.p. application of 30 μg CVF (16.2 IU) in PBS. Monitoring closely and using humane endpoint defining criteria survival was assessed. Data were combined from four independent identically performed experiments. Statistical analysis was performed by log-rank Mantel-cox test. Statistical significance according to the calculated p-value: *p ≤ 0.0332; **p ≤ 0.0021; ***p ≤ 0.0002; ****p ≤ 0.0001. [file Image_2.TIF]

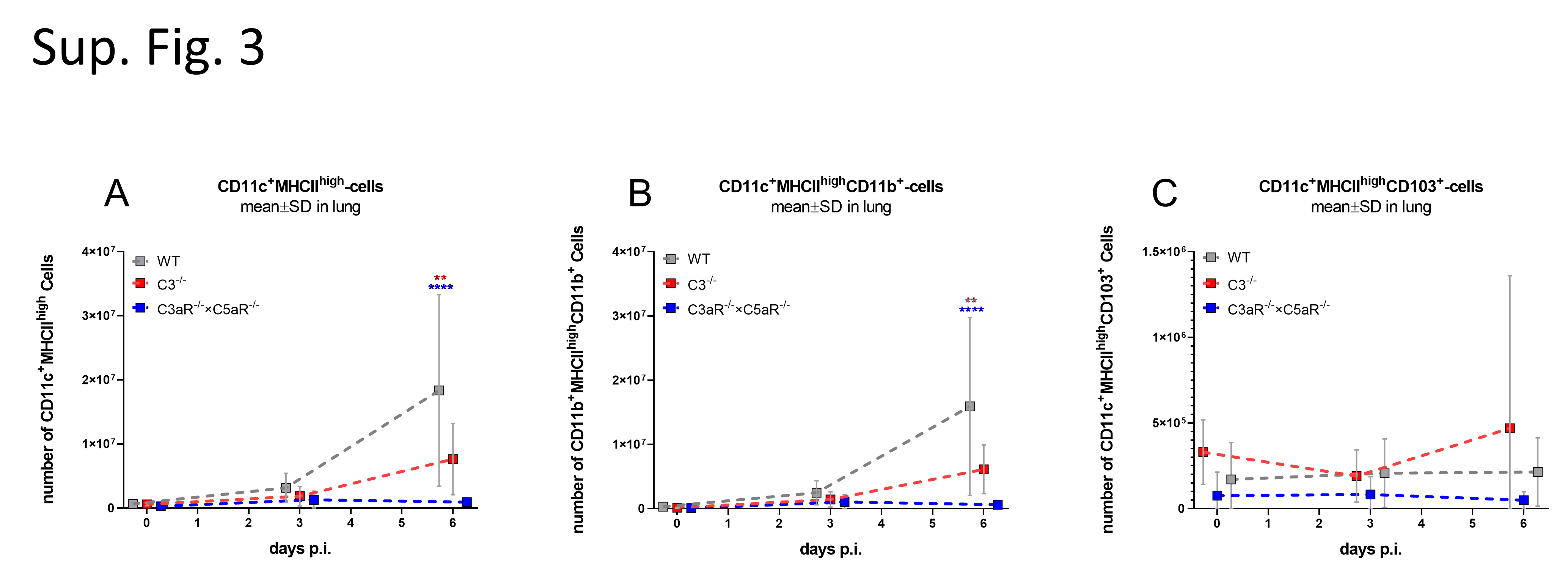

Supplement: Supplementary Figure 3 — Kinetics of the number of DCs in the mouse lung before and during the first days of C. psittaci infection in WT, C3−/− and C3aR−/− × C5aR1−/− mice. Amount of DCs in the lung immediately before and during C.ps. infection. Lung CD11c+-cells were purified by magnetic bead separation. DCs were identified as lineage-non auto fluorescent CD11c+MHCIIhigh-expressing cells and subtypes classified based on the expression of CD103+ or CD11b+, considering isotype and FMO controls. The numbers of CD11c+MHCIIhigh (A) CD11c+MHCIIhighCD11b+ (B) and CD11c+MHCIIhighCD103+ (C) in the lung were determined at day 0, 3, and 6 post C.ps. infection. Statistical analysis was performed by One-way ANOVA followed by Bonferroni post-test (n = 5–6 in each group and for each time-point; data were combined from four independent identical experiments). Statistical significance was displayed according to the calculated p-value (*p ≤ 0.0332; **p ≤ 0.0021; ***p ≤ 0.0002; ****p ≤ 0.0001). C.ps., Chlamydia psittaci; DC, dendritic cell; FMO, fluorescence minus one control. [file Image_3.TIF]
