# Supplementary material for: Complement and Chlamydia psittaci: Early Complement-Dependent Events Are Important for DC Migration and Protection During Mouse Lung Infection
Source: Front Immunol. 2021 Mar 9;12:580594. doi: 10.3389/fimmu.2021.580594 (PMC7986412; doi:10.3389/fimmu.2021.580594)
Supplement: Supplementary Table 1 — Clinical scoring. [file Data_Sheet_1.pdf]

Sup. Table 1.: Clinical scoring

Parameter	Clinical Observation	Points
Piloerection	Normal coated fur<br><br>Partial ruffed fur<br><br>Slightly ruffed fur<br><br>Ruffed fur	0<br><br>1<br><br>2<br><br>3
Body posture	Normal posture<br><br>Slightly hunched posture<br><br>Hunched posture<br><br>Heavily bent posture	0<br><br>1<br><br>2<br><br>3
Locomotion	Spontaneous movement, movement after cage opening or provocation of sleeping animals, normal behavior<br><br>Movement only after provocation<br><br>No movement – **humane endpoint**	0<br><br>1<br><br>2
Agility	Normal and fast movements<br><br>Slow and / or sluggish	0<br><br>1
Breathing	Unaffected<br><br>Tachypnea<br><br>Tachypnea with abdominal effort while breathing<br><br>Cyanotic oral mucus membranes, gasping for air – **humane endpoint**	0<br><br>1<br><br>2<br><br>3
Reaction to tactile stimulation	Normal attentiveness, escape reflex at approach<br><br>Attentiveness slightly affected, reduced reaction to external stimuli<br><br>Somnolent – **humane endpoint**	0<br><br>1<br><br>2
Ocular or nostril discharge	None<br><br>Presence of ocular and / or nostril discharge<br>(→ treatment with eye ointment)<br><br>Conjunctivitis with thick ocular discharge<br>(→ treatment with eye ointment)	0<br><br>1<br><br>2
Dehydration (exsiccosis)	Regular skin turgor<br><br>Reduced skin turgor, delayed spread of skin fold<br>(→ mashed and soaked wet food on cage floor)<br><br>Poor skin turgor, no spread of skin fold – **humane endpoint**	0<br><br>1<br><br>2
Body weight loss	0-3%<br><br>3-10% (→ mashed and soaked wet food on cage floor)<br><br>10-20% (→ mashed and soaked wet food on cage floor)<br><br>>20% for ≥ 10 continuous days – **humane endpoint**	0<br><br>1<br><br>2<br><br>3
Sum	Sum of clinical score ≥ 14 – **humane endpoint**<br><br>Control frequency: daily (during infection experiment)<br><br>Additional measures to increase animal well-being:<br><br>• All animals receive prophylactic rich breeding food (day 0)<br>• Clinical score ≥ 5: daily control frequency<br>• Clinical score ≥ 12 and / or ≥ 5 yellow criteria are met: control frequency of animals twice a day	Σ21

Sup. Table 2: Scoring system to evaluate the pathology of deparaffinized lung sections

Criteria	Score	Pathology
number of inflammatory cells	0	none, number of macrophages normal
	1	low number of inflammatory cells
	2	high number of inflammatory cells
degree of tissue bleeding	0	none
	1	low number of erythrocytes in alveoli
	2	high number of erythrocytes in alveoli
presence of oedema	0	none
	1	moderate
	2	extensive
affected areas	0	none
	1	0-10%
	2	10-20%
	3	25-50%
	4	50-75%
	5	>75%
peribronchial infiltrate	0	none
	1	low
	2	moderate
	3	evident
	4	extensive
	5	severe
luminal exudation	0	none
	1	low
	2	moderate
	3	evident
	4	extensive
	5	severe
overall pathology	0	none
	1	low, 10%
	2	moderate, 10-25%
	3	evident, 25-50%
	4	extensive, 50-75%
	5	severe >75%
